# Deep learning personalized recommendation-based construction method of hybrid blockchain model

**DOI:** 10.1038/s41598-023-39564-x

**Published:** 2023-10-20

**Authors:** Xiaomo Yu, Wenjing Li, Xiaomeng Zhou, Ling Tang, Rohit Sharma

**Affiliations:** 1 Guangxi Key Lab of Human-Machine Interaction and Intelligent Decision, Nanning Normal University, Nanning, 530001 Guangxi China; 2 Department of Logistics Management and Engineering, Nanning Normal University, Nanning, 530001 Guangxi China; 3 Arts Institute, Guangxi University for Nationalities, Nanning, 530001 Guangxi China; 4 Department of Electronics and Communication Engineering, SRM Institute of Science and Technology, NCR Campus, Modinagar, Ghaziabad, UP India

**Keywords:** Biological techniques, Energy science and technology

## Abstract

This study aims to explore the construction of a personalized recommendation system (PRS) based on deep learning under the hybrid blockchain model to further improve the performance of the PRS. Blockchain technology is introduced and further improved to address security problems such as information leakage in PRS. A Delegated Proof of Stake-Byzantine Algorand-Directed Acyclic Graph consensus algorithm, namely PBDAG consensus algorithm, is designed for public chains. Finally, a personalized recommendation model based on the hybrid blockchain PBDAG consensus algorithm combined with an optimized back propagation algorithm is constructed. Through simulation, the performance of this model is compared with practical Byzantine Fault Tolerance, Byzantine Fault Tolerance, Hybrid Parallel Byzantine Fault Tolerance, Redundant Byzantine Fault Tolerance, and Delegated Byzantine Fault Tolerance. The results show that the model algorithm adopted here has a lower average delay time, a data message delivery rate that is stable at 80%, a data message leakage rate that is stable at about 10%, and a system classification prediction error that does not exceed 10%. Therefore, the constructed model not only ensures low delay performance but also has high network security performance, enabling more efficient and accurate interaction of information. This solution provides an experimental basis for the information security and development trend of different types of data PRSs in various fields.

## Introduction

With the rapid development of artificial intelligence (AI) technology, all walks of life have produced massive data. While analyzing data information, various recommendation systems have also risen. In this trend, recommendation system extracts relevant data features after extracting user browsing information, and then achieves the recommendation effect of satisfying user interests. In the process of extracting users’ data information, it is very likely to cause irreparable losses to their personal property and social economy due to the leakage of user privacy information. Therefore, while using the AI algorithms such as deep learning (DL) to construct personalized recommendation system (PRS), data security has become the focus of scholars in related fields.

With the rapid development of AI technology, massive amounts of data are being generated across various industries. Along with the analysis of these data, various recommendation systems have emerged. These systems extract relevant data features by analyzing user browsing information and achieve the recommendation effect of satisfying user interests. However, in the process of extracting users’ data information, it is highly likely that the leakage of user privacy information could cause irreparable losses to their personal property and social economy. Therefore, while using AI algorithms such as deep learning (DL) to construct personalized recommendation systems (PRS), data security has become the focus of scholars in related fields.

DL-based PRS can capture the intricate internal relationships among massive amounts of collected data, leading to higher prediction accuracy compared to traditional statistics^1,2^. However, recommending users' personal habits, preferences, and browsing records through PRS may risk users’ personal sensitive data leakage due to limited storage capacity and network communication resources under the edge network. Thus, achieving data security and effective feature information extraction is of paramount practical significance. Blockchain, as a mechanism for data encryption, data chain distribution, multi-copy storage, and distributed consensus, can perform decentralized and distributed data management^3^. The main feature of blockchain is sub-block storage, where each block records the identity document of the previous block and contains some data^4^. This chain structure ensures the authenticity and immutability of data in each block^5^. With the development of blockchain technology, its unique incentive mechanism, and features of decentralization and non-tampering, it has found extensive applications in various fields such as supply chain, storage, and finance^6^. Moreover, blockchain technology leverages asymmetric encryption of public and private keys to encrypt, decrypt, store, and transmit data, thus holding great value in data privacy and security protection.

Ensuring the security of users’ data privacy while providing personalized services is becoming increasingly essential with the rapid development of mobile communication technology and the growing use of recommendation systems. In this study, a DL algorithm is introduced and optimized using the back propagation Levenberg–Marquardt (BP-LM) method. Additionally, the blockchain mechanism is improved, and a delegated proof of stake-byzantine algorand-directed acyclic graph (DPoS-BA-DAG) consensus algorithm suitable for public chains, namely the PBDAG consensus algorithm, is designed. Furthermore, a personalized recommendation model is constructed based on the hybrid blockchain PBDAG consensus algorithm and the optimized BP algorithm. Finally, the model's performance is analyzed through simulation, providing experimental references for improving the information security and classification accuracy of different types of data personalized recommendation systems in various fields.

## Related work

### Application status analysis of DL in recommendation systems

As the DL algorithm is being increasingly used in various fields, it has achieved remarkable results, particularly in the field of PRS, which has attracted the attention of scholars due to its advantages in prediction accuracy. Rosa et al.^7^ presented a Knowledge-based Recommendation System (KBRS) for sending warning messages to authorized persons when a monitoring system detects depression interference. The system detects sentences containing depression and stress content by using Convolutional Neural Network (CNN) and Bidirectional Long-Short-Term Memory-Recurrent Neural Network. The experimental results show that the proposed KBRS achieves a rating of 94%, with low memory, processing, and energy consumption of current mobile electronic devices. Huang et al.^8^ proposed an efficient passenger search recommendation framework using a multi-task DL paradigm. The framework contains two modules: Offline Training of Passenger-Hunting Recommendation Model and Online Application of Passenger-Hunting Recommendation Model. The results revealed that the recommended framework realizes the prediction of the area to pick up passengers, the degree of road congestion, and the carrier income, proving its feasibility and effectiveness. Iwendi et al.^9^ put forward a DL solution using health-based medical datasets that enables recommendation of patients’ diets according to disease and other characteristics. The results suggested that the Long Short-Term Memory technique is superior to other schemes in prediction accuracy, recall, accuracy, and F1 measure, and the recommended accuracy reaches 97.74%. Jin et al.^10^ proposed the application of a person in the circuit parallel learning framework and its end-to-end recommendation system that mimics and enhances the behavior of professional signal control engineers. Experimental evaluation revealed that the signal recommendation system proposed in this study can significantly improve traffic efficiency. Wen^11^ designed an intelligent background music system using DL and Internet of Things (IoT) technology and applied it in smart homes. Finally, the results revealed that the feature extraction algorithm under the middle layer feature construction has the highest recognition rate for indoor scenes, at about 87.6%, while the recognition rate of the Gabor feature algorithm for scene classification and recognition is generally about 20%.

The intelligent recommendation system is a widely used tool across various fields. Circular Neural Networks and CNNs in DL are commonly utilized to construct a recommendation system with high prediction accuracy. However, in the process of using these recommendation systems, the issue of data information leakage and data security concerns arise, particularly during data transmission and sharing. Such security vulnerabilities may cause irreversible losses to society or personal economy. Therefore, this study aims to address the problem of data security when constructing recommendation systems.

### Application analysis of blockchain

Blockchain technology has experienced several stages of application, including digital encryption money, financial industry, and practical scenarios. The mechanism of trust cooperation in distributed computing and computing environment is at the core of this technology, and it can solve challenges related to scalability, cooperation ability, trust relationship, and security protection in various application fields. Pradhan^12^ proposed a flexible permissioned ascription scheme using on-chain and off-chain licensing schemes via Orion and Metamask wallets. Contractual permissions were also utilized through a JavaScript-based chaincode deployed on Hyperledger Besu, which is an Ethereum-based permissioned blockchain network, and the Istanbul Byzantine Fault Tolerance 2.0 consensus algorithm was implemented. The proposed energy trading framework demonstrated efficient performance for deploying, transmitting, and querying energy transactions to a P2P energy trading blockchain network. Similarly, Wang et al.^13^ proposed a reputation-based consensus protocol for IoT systems supporting blockchain to improve IoT system security. The protocol used improved blockchain technology to resist attacks and allowed users with lower computing power more opportunities to join the consensus. The results indicated that the reputation-based consensus agreement provides advantages in safety and improved resistance to aggression. Himeur et al.^14^ investigated the horizontal challenges that arise when developing intelligent recommender systems, such as scalability, interpretability, distributed processing, and load balancing. These challenges present new research opportunities for blockchain developers and researchers. Kaur and Ali^15^ investigated the use of distributed computing in blockchain for enhancing the security of IoT systems. The study revealed that integrating blockchain into IoT frameworks significantly improves the overall security level compared to the IoT framework that does not employ blockchain. In another study, Sun et al.^16^ proposed an encrypted and searchable algorithm for querying intelligent traffic information in blockchain. This algorithm employs searchable encryption and the characteristics of blockchain to ensure the security and efficient query of logistics information. The logistics information is split into multiple data files, encrypted using asymmetric searchable encryption, and stored in a cloud server. The proposed scheme's correctness, completeness, and safety are evaluated, demonstrating its feasibility in practical applications.

Although blockchain technology has shown good results in addressing security issues in various fields, the potential problems that may arise in blockchain deployment schemes, such as node calculation, storage, network resource bottleneck, and scalability, have not been analyzed in detail. Therefore, this study aims to improve the traditional blockchain deployment scheme and construct a hybrid blockchain model that can significantly enhance its performance.

## Analysis of personalized recommendation model based on hybrid blockchain and DL algorithm

### Application analysis of the BP neural network algorithm in recommendation system

DL has been widely applied in recommendation systems, and there are many excellent algorithms. The general design idea is to convert discrete high-dimensional features into continuous features with a constant length through a feature embedding operation, and then extract features through multiple fully connected layers. Finally, the corresponding recommendation prediction probability is obtained through the activation of the corresponding activation function^17,18^. In this study, the recommendation system using the BP neural network is analyzed.

Equation (1) indicates the empirical equation for the number of hidden layers in three-layer neural networks.

1 $$h = \frac{1}{2}\left( {Y + O} \right) + a,a = 1,2, \ldots ,10$$

In Eq. (1), *Y* stands for the number of neurons; *O* signifies the number of neurons in the output layer; *a* is a constant, and the number of $h = 8 + a$ neurons is obtained according to the empirical equation. The activation function $\phi \left( x \right)$ of the network’s hidden layer is one of the necessary components to regulate neuronal activity^19^.

Furthermore, the weights and thresholds are analyzed. The connection weight from the second layer neuron *i* to the first layer neuron *j* is $w_{ij}$; the threshold of the second layer neurons is $\theta_{i}$; the connection weight of the third layer neurons *k* to the second layer neurons *i* is $w_{ki}$; the threshold of the third layer neurons is $a_{k}$. Then, the model objective can be expressed as Eq. (2).

2 $$E = \frac{1}{2}\sum\limits_{p = 1}^{P} {\sum\limits_{k = 1}^{L} {\left( {T_{k}^{p} - O_{k}^{p} } \right)^{2} } }$$

The trained neural network can obtain the ideal output value by approximating new input; that is, the error between the actual output and the ideal output is minimized.

After applying the BP-LM method, the input data is propagated forward and backward through the neural network. The forward propagation fits the input data to the output of the network, but only a trained network can produce accurate results. Hence, the network is trained by utilizing BP to provide feedback on the error between the actual and expected outputs of the model, and to adaptively adjust the weight and threshold of each neuron. The trained neural network can effectively fit the input data ^20^.

During data analysis, each neuron in the input layer influences each neuron in the hidden layer, and the input of the *i*-th neuron in the hidden layer can be calculated via Eq. (3).

3 $$YSR_{i} = \sum\limits_{j = 1}^{13} {w_{ij} Y_{j} + \theta_{i} }$$

Equation (4) describes the output $YSC_{i}$ of the *i*-th neuron in the hidden layer.

4 $$YSC_{i} = \phi \left( {YSR_{i} } \right) = \phi \left( {\sum\limits_{j = 1}^{13} {w_{ij} Y_{j} + \theta_{i} } } \right)$$

The input $SCSR_{k}$ at the *k* node of the output layer is presented in Eq. (5).

5 $$SCSR_{k} = \sum\limits_{i = 1}^{13} {w_{ki} YSC_{i} + a_{k} } = \sum\limits_{i = 1}^{13} {w_{ki} \phi \left( {\sum\limits_{j = 1}^{13} {w_{ij} Y_{j} + \theta_{i} } } \right) + a_{k} }$$

Its output $o_{k}$ is expressed as Eq. (6).

6 $$o_{k} = \psi \left( {SCSR_{k} } \right) = \psi \left( {\sum\limits_{i = 1}^{13} {w_{ki} YSC_{i} + a_{k} } } \right) = \psi \left( {\sum\limits_{i = 1}^{13} {w_{ki} \phi \left( {\sum\limits_{j = 1}^{13} {w_{ij} Y_{j} + \theta_{i} } } \right) + a_{k} } } \right)$$

When classifying data, issues such as unclear classification or information leakage may still arise. Blockchain technology provides a mechanism that utilizes encryption algorithms, multi-copy storage, and distributed consensus to ensure that privacy data sharing remains uncompromised. The technology can balance data monopoly and optimize industry classification models by carrying out data mining on encrypted data.

### Improvement and analysis of blockchain technology

**Figure 1:**
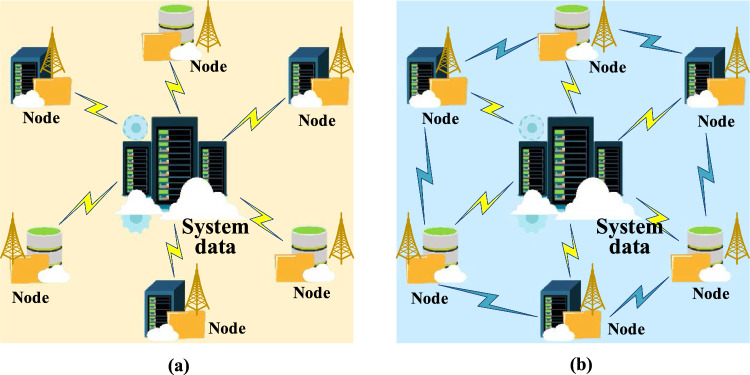
Comparison of data storage and recording methods between centralized and distributed systems (**a**. centralized; **b**. distributed) (The picture comes from the author's own drawing, inspired by: https://www.shutterstock.com/zh/image).

Blockchain literally consists of “block” and “chain” and is a technology that was adopted at the foundation of Bitcoin, also known as a hash chain^21^. From a basic principle perspective, a block is the data generated by legitimate transactions within a certain time frame that are packaged by miners who calculate the hash difficulty. It is a format for storing data units, where each transaction is a data unit. The chain refers to each block containing the hash index of the previous block, and the block is broadcasted by a distributed network composed of a point-to-point network. Upon receiving a new block, each node will compare it with their existing block data. If the block is not yet included and its validity is verified, it will be updated to the longest blockchain data^22^. Its accounting model is relative to the traditional central model and uses a decentralized distributed network of nodes to record data together (Fig. 1).

**Figure 2:**
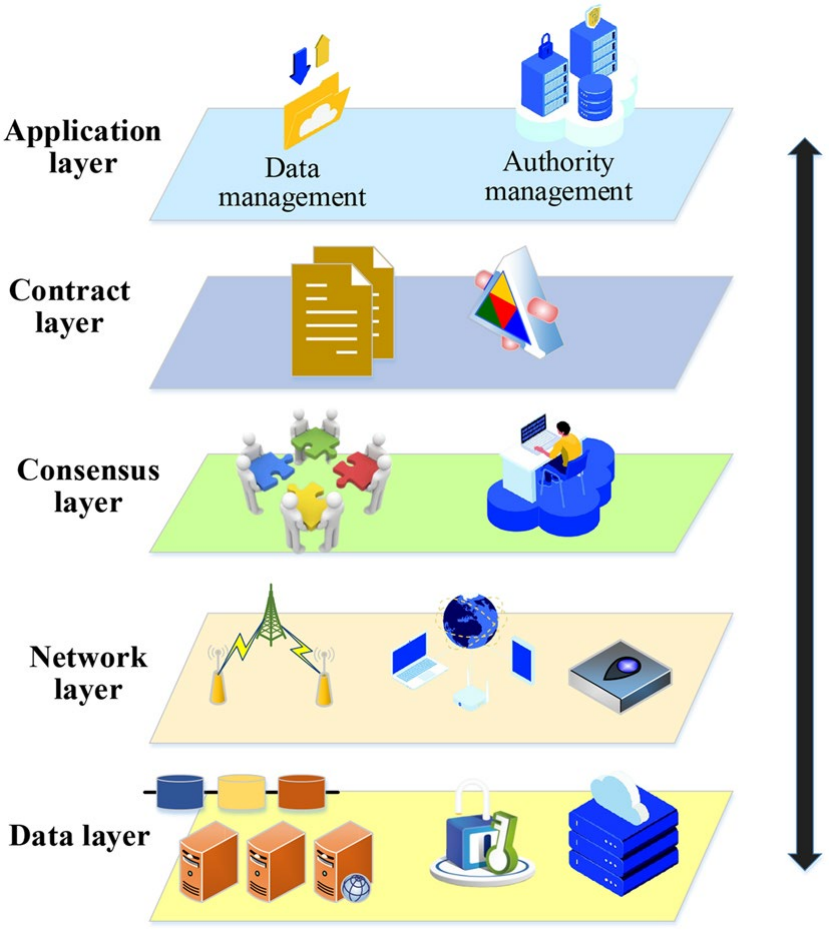
Distributed blockchain system (the picture comes from the author's own drawing, inspired by: https://www.shutterstock.com/zh/image).

With the continuous development of blockchain technology, it can be divided into three categories: public chain, alliance chain, and private chain. Moreover, as a distributed blockchain system, Ethereum provides the concept of smart contracts. The scripting language of smart contracts can be run on the Ethereum Virtual Machine, which is a decentralized virtual machine that executes smart contracts on the Ethereum network^23^. The structure of Ethereum is mainly composed of six layers: the application layer, smart contract layer, incentive layer, consensus layer, network layer, and data layer (Fig. 2).

The diagram in Fig. 2 depicts the architecture of the Ethereum blockchain platform. The application layer utilizes the distributed computing platform of Ethereum to provide programmable currency and finance based on smart contracts. The contract layer serves as the foundation of Ethereum’s smart contract and is a well-developed scripting language that interacts with both the upper application and lower data layers. The consensus layer ensures consensus on the data in the block in a fully distributed environment. The network layer is responsible for node connection, transaction broadcasting, and block broadcasting. The data layer is the foundation of the data structure in Ethereum and includes hash functions, asymmetric encryption, and other encryption algorithms. High-quality data sharing across industries is promoted by combining the classification algorithm, encryption scheme, and blockchain smart contract.

During the digital trading process, the private key is used to sign each transaction digitally, ensuring that unauthorized parties cannot tamper with the transaction, and that the transaction is initiated by the sender^24,25^. Let *d* be the private key and *g* be the circular point (x, y). Then, the public key is expressed as Eq. (7) according to the elliptic curve algorithm:

7 $$q = d \times g$$

In the process of transaction signature and verification in digital signature algorithm, the signature process requires the sender’s private key to encrypt the transaction.

*Step 1* the random number $k \in \left[ {1,n - 1} \right]$ is selected, and according to Eq. (8), the point $\left( {x_{1} ,y_{1} } \right)$ is calculated.

8 $$\left( {x_{1} ,y_{1} } \right) = k \times g$$

*Step 2* *r* is calculated based on Eq. (9). If *r* = 0, return the first step to reselect the random number *k*.

9 $$r = x_{1} \bmod n$$

*Step 3* if $r \ne 0$, the hash value $h\left( m \right)$ of message digest *m* is computed according to Eq. (10).

10 $$h\left( m \right) = HASH\left( m \right)$$

*Step 4* $S_{v}$ is calculated based on *k*, $h\left( m \right)$, private key $SKv$, and Eq. (5). If $S_{v} = 0$, return to the first step to reselect the random number *k*.

11 $$S_{v} = \left[ {\left( {h\left( m \right) + SKv*r} \right)*k^{ - 1} } \right]\bmod n$$

*Step 5* if $S_{v} \ne 0$, the signature of $h\left( m \right)$ is $\left\{ {r,S_{v} } \right\}$.

The original information is further validated.

*Step 1* if $\left\{ {r,S_{v} } \right\}$ are integers between $\left[ {1,n - 1} \right]$, Eq. (12) holds.

12 $$\omega = S_{v}^{ - 1} \bmod n$$

*Step 2* the message digests *A* and *B* are computed.

13 $$\mu_{1} = \left( {HASH\left( m \right) \cdot \omega } \right)\bmod n$$

14 $$\mu_{2} = \left( {r \cdot \omega } \right)\bmod n$$

*Step 3* a point on an elliptic curve is computed.

15 $$\left( {x_{1} ,y_{1} } \right) = \mu_{1} g + \mu_{2} q$$

*Step 4*, solve Eq. (16).

16 $$r = x_{1} \bmod n$$

If *r* equals 0 or ∞, the signature is rejected; otherwise, the verification passes.

**Figure 3:**
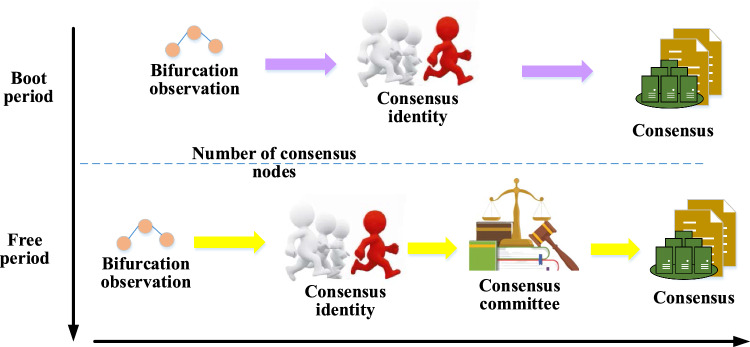
Flow of the PBDAG consensus algorithm (this picture comes from the author's own drawing, inspired by: Literature^26,27^).

It is essential to improve its application to address the limitations of basic blockchain technology, such as limited node computing power, rapid storage consumption growth, and poor scalability. This study focuses on improving blockchain by developing a consensus algorithm called PBDAG for the public chain. The consensus process of the PBDAG algorithm is illustrated in Fig. 3. The hybrid blockchain model can enhance the reliability and security of personalized recommendation systems based on deep learning, by incorporating the PBDAG consistency algorithm. The PBDAG algorithm, originally designed for sequence assembly of multiple DNA reads, can verify data consistency in the hybrid blockchain model. By establishing a branching tree based on the Directed Acyclic Graph (DAG) data structure and removing branches that violate consistency principles, the PBDAG algorithm yields a relatively accurate genome sequence. Blockchain technology provides a tamper-proof record of user behavior data and recommendation algorithm models. Smart contracts running on the blockchain can ensure access control and data management, thus safeguarding user data. The PBDAG algorithm gradually identifies and removes branches that do not conform to the consistency principle to ensure the accuracy and consistency of the assembled sequence. This approach improves the reliability and security of personalized recommendation systems under the hybrid blockchain model.

In the blockchain domain, the PBDAG algorithm is commonly used as a consensus algorithm to verify the legality of transaction data and ensure data consistency and reliability in the network. Compared with traditional consensus algorithms, the PBDAG algorithm has the advantage of requiring less storage space and computational complexity. By using the PBDAG algorithm, users' suggestions and evaluations can be verified, thus preventing malicious users from manipulating data and influencing the accuracy of the recommendation algorithm. In addition, the PBDAG algorithm can also be utilized for the consensus mechanism of the hybrid blockchain model. Since the PBDAG algorithm uses a strategy of identifying and removing branches that violate the consistency principle to achieve genome splicing, it can be applied to consistency checking and consensus among nodes in the blockchain. Specifically, the PBDAG algorithm can identify and remove inconsistent data among nodes to ensure the blockchain network achieves a consistent state. Additionally, the consensus mechanism based on the PBDAG algorithm can use the contribution of network participants as a reference evaluation of authority and reliability on the blockchain network.

As depicted in Fig. 3, the proposed consensus algorithm consists of a piecewise consensus process. As the number of consensus nodes in the model increases, it can be divided into two periods, namely the guiding period $\left( {E_{boot} } \right)$ and the free period $\left( {E_{freedom} } \right)$. Different consensus algorithms are used in each period, where DLattice-Practical Byzantine Fault Tolerance (PBFT) consensus is employed in the guiding period, and DLattice-PBDAG consensus is adopted in the free period. The nodes participating in the guiding period are referred to as guidance nodes. The transaction blocks in the model are denoted as $List_{TB} = \left\{ {TB_{send} ,TB_{send}^{\prime } ,TB_{send}^{\prime \prime } , \ldots ,TB_{send}^{n} } \right\}$, and each node observes the bifurcation transaction set $\left\{ {TB_{send} ,TB_{send}^{\prime } ,TB_{send}^{\prime \prime } , \ldots ,TB_{send}^{n} } \right\}$, resulting in a bifurcation. After observing the bifurcation, the candidate consensus node starts to calculate a legitimate consensus identity to participate in the consensus and resolve the bifurcation. During the guiding period $\left( {E_{boot} } \right)$, each candidate consensus node $\left( {Candidates_{Seed} } \right)$ has a unique consensus identity at each stage of the consensus.

17 $$ID_{Seed} \leftarrow \left\langle {P,Seed,Sig_{pk} } \right\rangle$$

In the free period $E_{freedom}$, $Candidates_{Seed}$ in the model secretly calculates the hash value satisfying the Proof of Stake condition locally.

18 $$hash \le w_{i} /W$$

In Eq. (18), $w_{i}$ refers to the sum of the voting weight held and represented by this node, that is, the interests of the node (Stake); $W$ signifies the total voting weight of the model. With other information such as its public key, it forms a legal identity to this consensus.

19 $$ID_{pos} \leftarrow \left\langle {hash,proof,message} \right\rangle$$

20 $$ID_{Seed} \leftarrow \left\langle {P,Seed,ID_{pos} ,Sig_{pk} } \right\rangle$$

**Figure 4:**
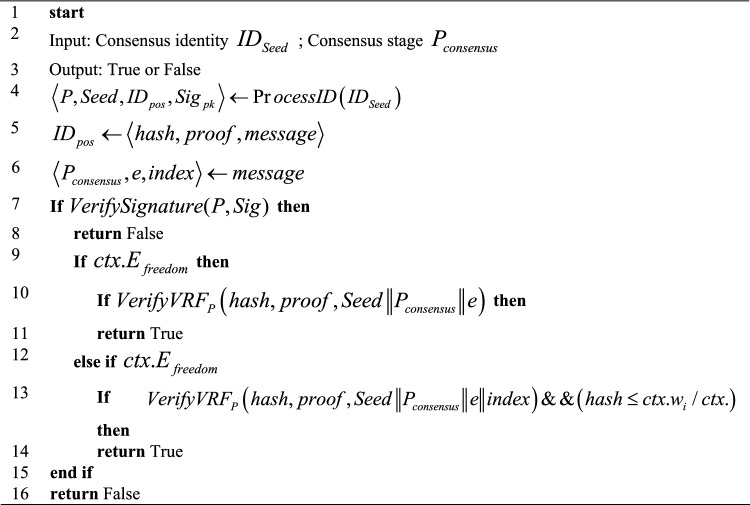
Algorithm flow for validation.

The aforementioned parameters collectively determine the calculation complexity of the consensus identity. In addition, nodes with higher stakes will generate more identities under the same calculation attempts, thereby increasing the likelihood of incurring a Fork Penalty. Upon completion, the identity undergoes verification, and the algorithm flow is presented in Fig. 4.

### Construction of the personalized recommendation model based on hybrid blockchain combined with the BP neural network algorithm

**Figure 5:**
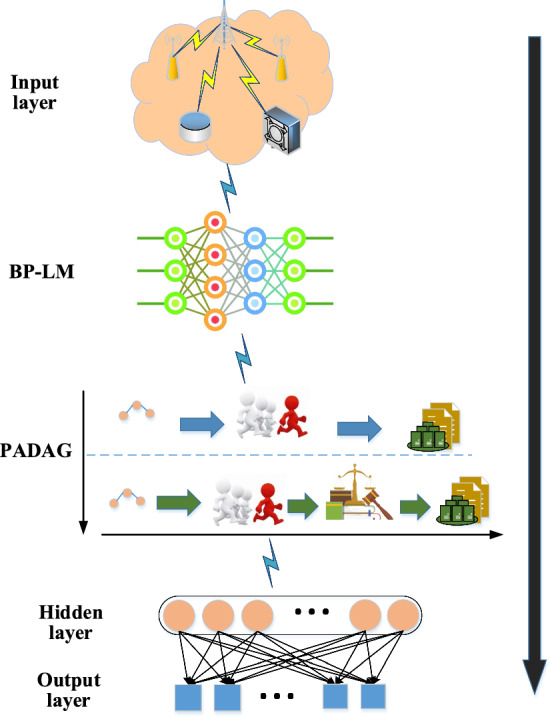
Personalized recommendation model based on hybrid blockchain PBDAG consensus algorithm combined with optimized BP algorithm (The picture comes from the author's own drawing, inspired by: Literature ^26,27^, and https://www.shutterstock.com/zh/image).

Given the current trend of generating massive data in personalized recommendation systems, protecting user privacy is of utmost importance. This study proposes a personalized recommendation system that utilizes the BP neural network algorithm and improved blockchain technology. The system is built using a hybrid blockchain PBDAG consensus algorithm combined with an optimized BP algorithm, as shown in Fig. 5.

As illustrated in Fig. 5, the personalized recommendation model reported here operates on the following assumptions:

#### Hypothesis 1:

Honest nodes in the permissioned blockchain run secure and dependable model software, and the percentage of tokens held by honest users is greater than the threshold h (a constant greater than 2/3). Malicious nodes can participate in the permissioned blockchain and hold some tokens.

#### Hypothesis 2:

There are at least 2f. + 1 honest nodes in the permissioned blockchain, where *f* represents the number of malicious nodes (Byzantine nodes) in the permissioned blockchain, and the total number of nodes in the permissioned blockchain is at least 3f. + 1.

#### Hypothesis 3:

Information transmitted by honest users in the permissioned blockchain can be received by other honest users within a certain time, and network partition is prohibited.

The security performance of the improved consensus algorithm is further analyzed in terms of its ability to prevent potential attacks and its ability to operate securely in the presence of malicious nodes.

In the generation stage of consensus identity in period $E_{freedom}$, the probability of generating each identity is determined by the Stake held by its nodes. The probability of generation and the number of attempts to calculate follow the exponential distribution^28,29^. It is assumed that $\beta$ represents the number of attempts required to calculate the identity, and *θ* represents the probability of calculating it once. The probability of obtaining a legitimate consensus identity within $\beta$ attempts is calculated via Eq. (21).

21 $$P\left\{ {x \le \beta } \right\} = 1 - P\left\{ {x - \beta } \right\} = 1 - \left( {1 - \theta } \right)^{\beta } = 1 - e^{{\beta \log \left( {1 - \theta } \right)}}$$

Giving $\theta \ll 1$, $\log \left( {1 - \theta } \right) \approx - \theta$, Eq. (22) can be obtained.

22 $$P\left\{ {x \le \beta } \right\} \approx 1 - e^{ - \theta \beta }$$

Therefore, the possibility and the number of calculations required meet exponential distribution. If $\theta = w_{i} /W$, $w_{i}$ is the node weight, and $W$ means the total weight of the model. Then, there is Eq. (23).

23 $$P\left\{ {x \le \beta } \right\} \approx 1 - e^{{\frac{{w_{i} \beta }}{W}}}$$

When $W = DLT_{total} = 12000DLT$, the weights of voting held by consensus nodes is $w_{i}$ = 10 *DLT* and $w_{j}$ = 20*DLT*, respectively, and the probability are different when the number of attempts to calculate is different. It can be found that for a given $\beta$, as the weight of voting increases, the probability of generating a valid consensus identity also increases.

The threshold of honest identity in the consensus committee (CC) is further analyzed. In the formation stage of the CC in the free period, candidate consensus nodes generate consensus identity and form a CC according to their Stake. The model ensures that the malicious identity in the CC cannot hinder the consensus formation^16^. The consensus account$w_{i}$ can generate *k* consensus identity probabilities $P\left\{ {x = k} \right\}$ in the $\beta$ calculation, based on its Stake.

24 $$P\left\{ {x = k} \right\} = \left( {\begin{array}{*{20}c} \beta \\ k \\ \end{array} } \right)\left( {\frac{{w_{i} }}{W}} \right)^{k} \left( {1 - \frac{{w_{i} }}{W}} \right)^{\beta - k}$$

Its expectation is $E = \beta w_{i} /W$. The pass $DLT_{good}$ held by honest nodes and the total system pass $DLT_{total}$ satisfy Eq. (25).

25 $$h = DLT_{good} /DLT_{total}$$

If *W* at consensus equals *DLT*_*total*_, the weight held by honest nodes is $W_{honest} = DLT_{good}$, and the weight held by malicious nodes is $w_{adversary} = DLT_{bad}$. The expectation of the number of consensus identities generated by honest and malicious nodes is shown in Eqs. (26) and (27).

26 $$E_{good} = \beta w_{honest} /W$$

27 $$E_{bad} = \beta w_{adversary} /W$$

Finally, the expected total number of consensus identities in the model is $C_{E} \approx \beta$. Equation (28) describes the relationship between the threshold $ID_{good}$ of honest identity in the CC, the maximum total number of identities $all_{\max }$, the minimum number of honest identities $h_{\min }$, and the maximum number of malicious identities $a_{\max }$ when the number of calculations $\beta$ is 100, 200, 300, 400, and 500, respectively, under different security parameter $\lambda$.

28 $$\left\{ {\begin{array}{*{20}c} {ID_{good} > 2 \times a_{\max } } \\ {ID_{good} \le h_{\min } } \\ {2 \times ID_{good} > all_{\max } } \\ \end{array} } \right.$$

If the threshold $ID_{good}$ meets honest identity in the CC, the only consensus can be reached in the consensus process.

### Experiment evaluation

The performance analysis of the model is conducted by deploying 50 to 500 non-licensed chain nodes in 25 virtual servers on Ali Cloud with 4 vCPUs and 8 GB memory. The implementation of a prototype system of non-licensing chain DLattice based on PBDAG consensus is carried out using Golang language. The P2P network is implemented using the go-libp2p library provided by IPFS, based on the Gossip protocol. The blockchain data are stored in Leve1DB as the backend database, and the original data are stored in IPFS. The model is developed on the Ethereum virtual machine platform for smart contract development. The hash function used in this study is SHA-256, and asymmetric encryption is applied using Elliptic Curve Integrated Encryption Schema^30^. The Elliptic Curve Digital Signature Algorithm is adopted, and the VRF function described by Goldberg is used^31,32^. Proxy Re-Encryption technology and AFGH algorithm are utilized for access management. The simulation parameters used in the implementation of the non-licensing chain DLattice prototype system in the evaluation of the model experiment are shown in Table 1.

**Table 1:** Simulation parameters used in the implementation of the non-licensing chain DLattice prototype system during the experimental evaluation process.

Parameter	Meaning	Value
$\lambda$	safety parameters	10
*h*	The ratio of the number of tokens held by honest users to the total	4:5
$DLT_{total}$	Total number of model tokens	1300
$DLT_{init}$	The number of tokens initially allocated by the model bootstrap node	65
$C_{B}$	size of steering committee	250
$C_{E}$	Desired number of consensus identities	250
$\tau_{good}^{{}}$	Proportion of honest identities to total identities during bootstrapping consensus	2:3
$ID_{good}^{{}}$	Consensus voting threshold for honest identity in free periods	125

The model presented here is based on the Ethereum Virtual Machine platform, which is a blockchain-based smart contract platform. The Ethereum Virtual Machine enables the execution of smart contract code on the blockchain, allowing the development of automated contracts that can execute transactions and protocols without the need for third-party intervention. Decentralized applications can be built using the Ethereum Virtual Machine and smart contracts, enabling applications to be maintained and operated by nodes on the blockchain network, rather than relying on centralized servers or third-party institutions. The use of the Ethereum Virtual Machine and smart contracts can improve the transparency, fairness, and security of the execution process of decentralized applications. It can also mitigate the risks associated with centralized institutions and single-point failures. Moreover, smart contracts can automatically execute without human intervention, enhancing the efficiency and reliability of applications. In this paper, the Ethereum Virtual Machine platform and smart contracts were chosen to construct the model to enhance its credibility and security.

To evaluate the performance of the model proposed here, it is compared with several widely used consensus algorithms such as Practical Byzantine Fault Tolerance (PBFT)^33^, Byzantine Fault Tolerance (BFT)^34^, Hybrid Parallel Byzantine Fault Tolerance (HPBFT)^35^, Redundant Byzantine Fault Tolerance (RBFT)^36^, and Delegated Byzantine Fault Tolerance (DBFT)^13^ in terms of recommendation accuracy and data security transmission performance. Three repeated experiments were performed to ensure the accuracy of the results.

## Results and discussion

### Accuracy analysis of PRS

The PBFT, BFT, h PBFT, RBFT, and DBFT consensus algorithms are implemented through code simulation. PBFT algorithm divides participants into clients, backup nodes, and primary nodes, and utilizes three stages of message exchange to achieve consensus results. On the other hand, the BFT algorithm implements a completely decentralized network where all nodes have the same status, without primary or backup nodes. To improve system scalability and fault tolerance, h PBFT combines the advantages of PBFT and BFT algorithms by dividing participants into two types of nodes: BFT nodes and PBFT nodes. Similarly, RBFT combines BFT and PBFT algorithms to enhance the fault tolerance of the system. The DBFT algorithm divides nodes in the network and delegates the consensus process to specific nodes, where only bookkeepers can participate in the consensus process. During the actual implementation process, code must be written according to the corresponding consensus protocol, and the corresponding network environment should be simulated to mimic message exchange and consensus process among nodes. Moreover, communication overhead and security issues between nodes must be considered to ensure the accuracy and reliability of the consensus results.

**Figure 6:**
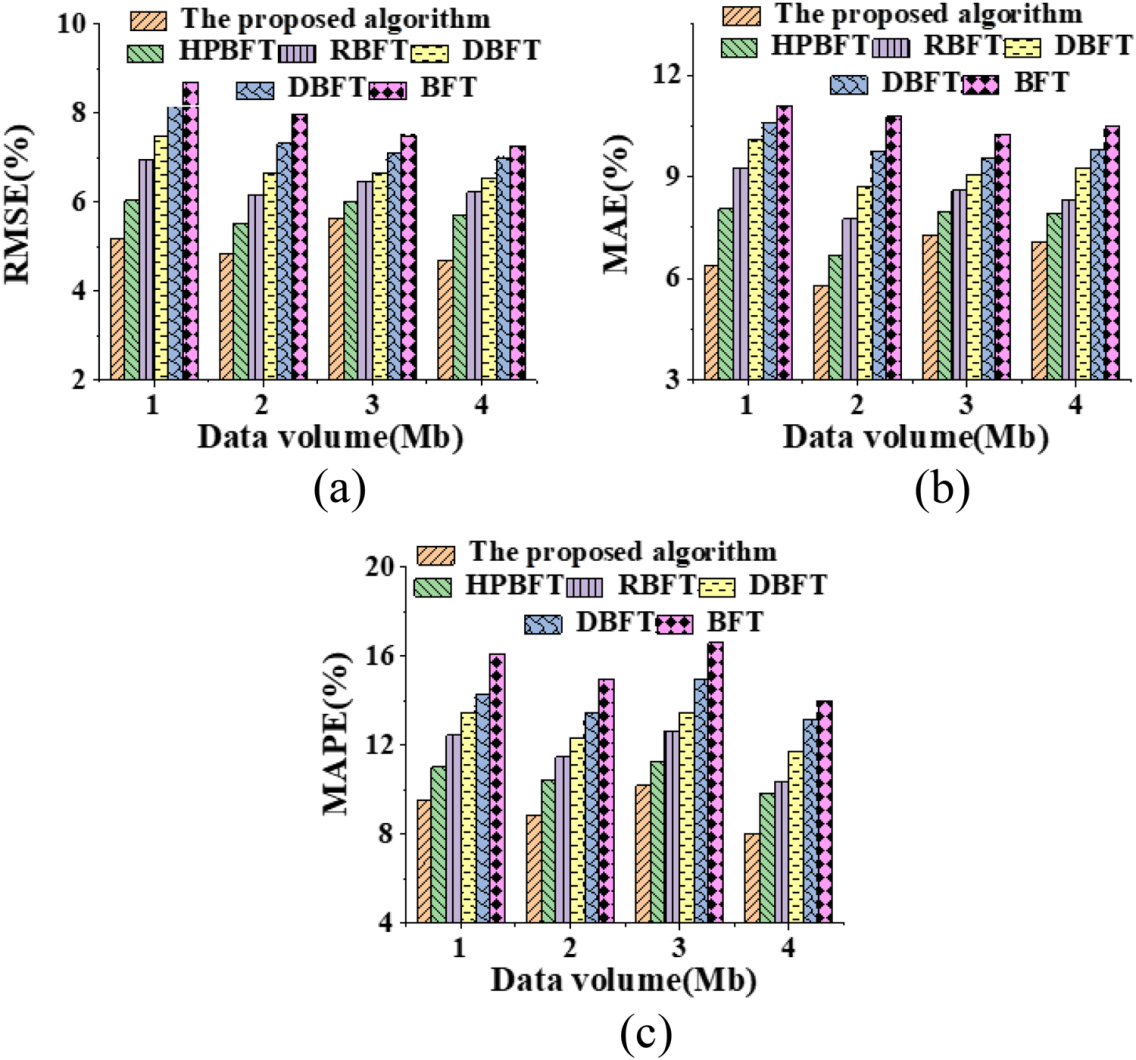
Different errors (%) of different mechanism algorithms with the increase of data volume (**a**) RMSE; (**b**) MAE; (**c**) MAPE.

The classification accuracy of the PRS reported here is evaluated by comparing it with PBFT, BFT, HPBFT, RBFT, and DBFT using RMSE, MAE, and MAPE metrics. The results are presented in Fig. 6 and show how the hybrid blockchain mechanism performs in terms of classification accuracy.

The findings are presented in Fig. 6. The comparison between the proposed hybrid blockchain PBDAG consensus algorithm with optimized BP algorithm and other algorithms used in previous research for personalized system recommendations is shown. The average RMSE, MAE, and MAPE are found to be 4.87%, 6.91%, and 8.68%, respectively, which are lower than the error values of other model algorithms. Thus, the personalized recommendation model algorithm based on the hybrid blockchain PBDAG consensus algorithm with optimized BP algorithm can significantly decrease the classification error of various types of data in the PRS and achieve a more accurate classification prediction effect.

### Data security transmission performance analysis of PRS with different mechanism algorithms

**Figure 7:**
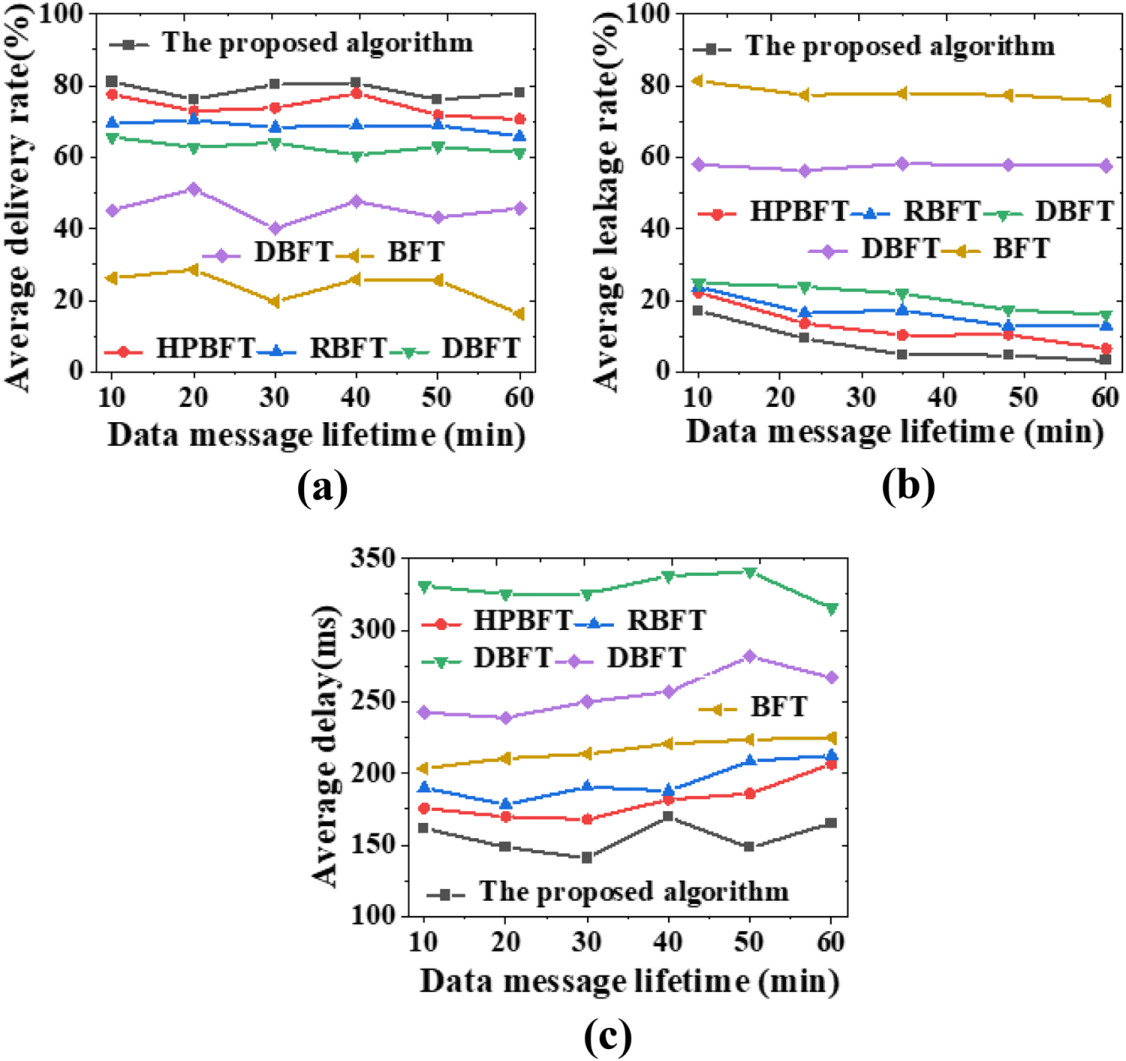
Comparison curve of security transmission of algorithms under different data message survival time (**a**) average delivery rate; (** b**) average leakage rate; (**c**) average delay.

**Figure 8:**
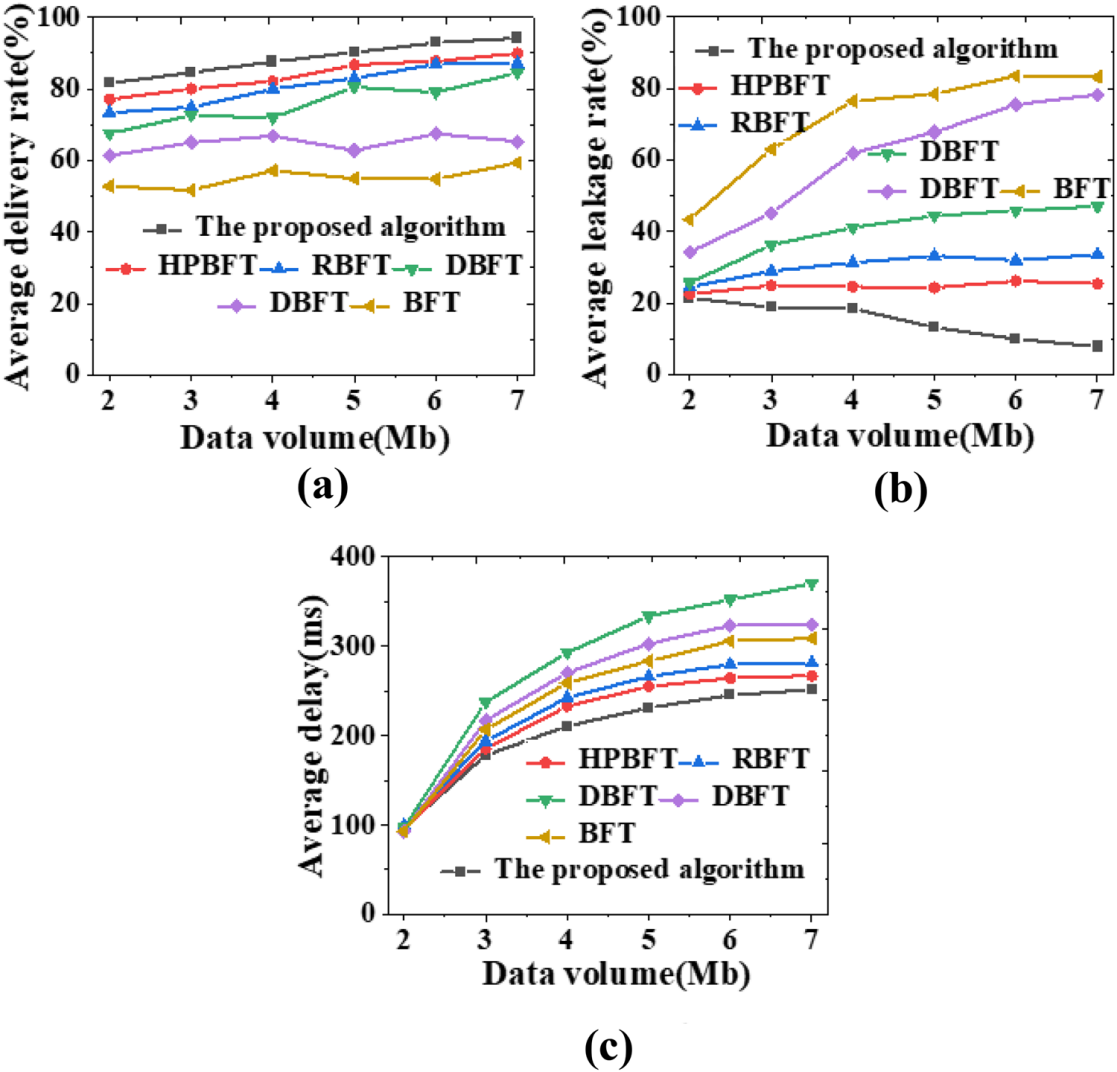
Comparison of network data security transmission of various mechanism algorithms under different data volumes (**a**) average delivery rate; (**b**) average leakage rate; (**c**) average delay.

**Figure 9:**
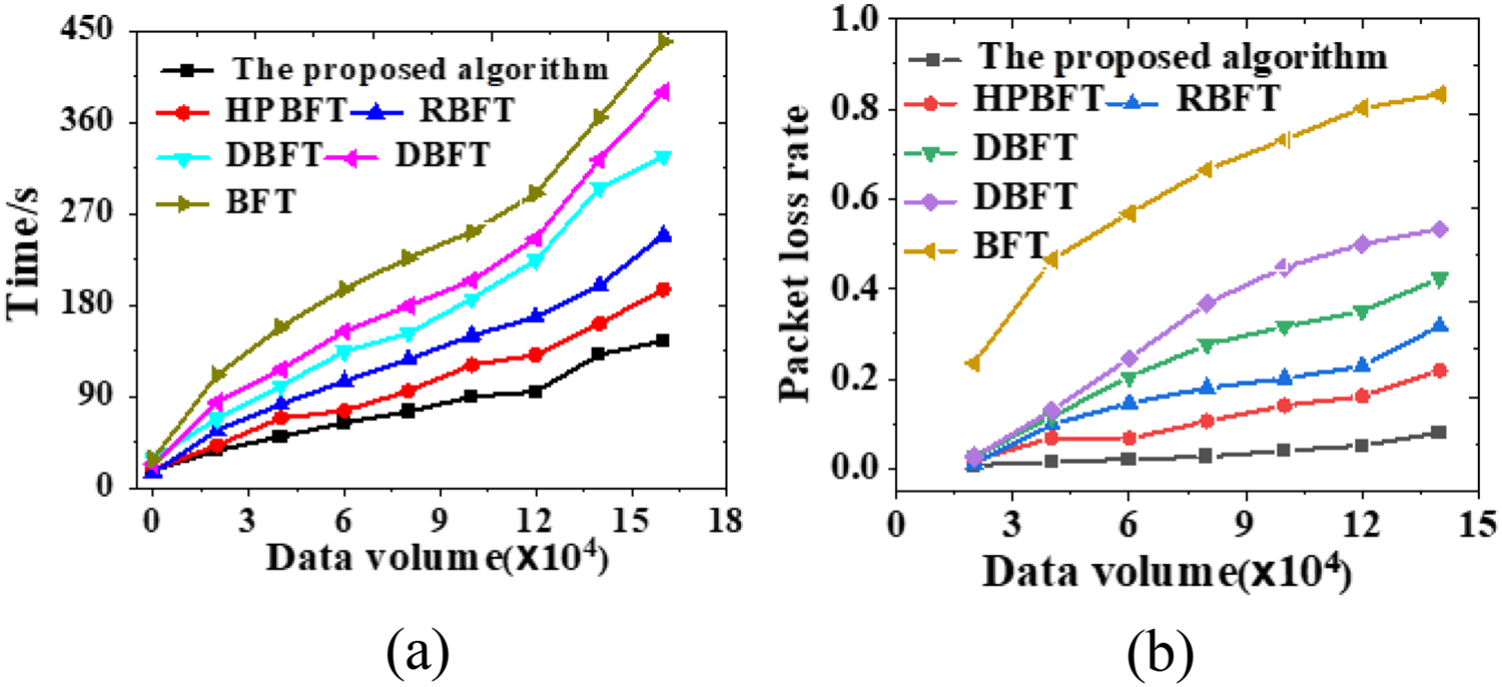
Comparison curve of time and packet loss rate required by various mechanism algorithms under different data volumes (**a**) required time; (** b**) packet loss rate.

The security of data transmission in the PRS model developed in this study is assessed by comparing the hybrid blockchain mechanism with other consensus algorithms, namely PBFT, BFT, HPBFT, RBFT, and DBFT. The comparison is based on different data volumes and data message survival time. The results of this analysis are presented in Figs. 7, 8, and 9.

As the survival time of data messages increases, the PRS model algorithm shows an improvement in delivery rate and a decrease in leakage rate. This could be due to the message fragmentation and trusted end-user forwarding strategy employed by the model algorithm, which ensures secure message transmission. In contrast, other algorithms may not have such robust security measures, or may only encrypt messages without proper trust evaluation of forwarding nodes. The average delay of the PRS model algorithm rises gradually with the survival time of the epidemic prevention and control data message, with the final average delay being around 150 ms.

As the amount of data transmitted by the system increases, the delivery rate of network data shows an increasing trend, and the delivery rate of data messages in this study is consistently above 80% (Fig. 8a). The average leakage rate does not show significant changes and is below 10% throughout the increasing data volume (Fig. 8b). Moreover, as the data volume increases, the average delay decreases, and the average delay of the model algorithm proposed here is about 350 ms. The model algorithm shows a higher average delivery rate, a lower average leakage rate, and a lower delay, indicating good network data security transmission performance.

The model algorithm reported here presents superior performance compared to other algorithms adopted by other researchers, especially in dealing with large data volumes in PRS scenarios, with a less sensitive response to data growth and more obvious acceleration effect. Moreover, the data loss rate of the algorithm does not increase significantly even with the increase of data volumes, staying below 0.1. Therefore, the personalized recommendation model based on the hybrid blockchain PBDAG consensus algorithm combined with the optimized BP algorithm still maintains better data transmission performance, as shown in the comparative analysis of the experimental results of all methods.

## Conclusion

Effective and secure extraction of feature information is crucial for various practical applications. This study introduces and improves blockchain technology to design a PBDAG consensus algorithm suitable for public chains. The algorithm is combined with an optimized BP algorithm to construct a personalized recommendation model based on the hybrid blockchain PBDAG consensus algorithm. Experimental evaluation shows that the model achieves a stable data message delivery rate of 80%, a leakage rate of approximately 10%, and a system classification prediction error of no more than 10%. The proposed algorithm can be applied to various types of data in different fields, providing an experimental foundation for enhancing the system's information security and classification accuracy. However, the algorithm has some limitations. For instance, in the consensus algorithm PBDAG, the corresponding membership of the CC is exposed and vulnerable to Distributed Denial of Service (DDoS) attacks if nodes fail to reach consensus in the first term. Although the affected CC only impacts the consensus of the corresponding bifurcation, avoiding DDoS attacks on the members remains a future research focus.

## Data Availability

Data sharing is not applicable to this article as no new data were created or analyzed in this study.
